# Combined model of radiomics and clinical features for differentiating pneumonic-type mucinous adenocarcinoma from lobar pneumonia: An exploratory study

**DOI:** 10.3389/fendo.2022.997921

**Published:** 2023-01-16

**Authors:** Huijun Ji, Qianqian Liu, Yingxiu Chen, Mengyao Gu, Qi Chen, Shaolan Guo, Shangkun Ning, Juntao Zhang, Wan-Hu Li

**Affiliations:** ^1^ Department of Radiology, Shandong Cancer Hospital and Institute, Shandong First Medical University and Shandong Academy of Medical Sciences, Jinan, Shandong, China; ^2^ GE Healthcare, Precision Health Institution, Shanghai, China

**Keywords:** pneumonic-type mucinous adenocarcinoma, lung cancer, lobar pneumonia, adenocarcinoma, inflammation, computed tomography

## Abstract

**Purpose:**

The purpose of this study was to distinguish pneumonic-type mucinous adenocarcinoma (PTMA) from lobar pneumonia (LP) by pre-treatment CT radiological and clinical or radiological parameters.

**Methods:**

A total of 199 patients (patients diagnosed with LP = 138, patients diagnosed with PTMA = 61) were retrospectively evaluated and assigned to either the training cohort (*n* = 140) or the validation cohort (*n* = 59). Radiomics features were extracted from chest CT plain images. Multivariate logistic regression analysis was conducted to develop a radiomics model and a nomogram model, and their clinical utility was assessed. The performance of the constructed models was assessed with the receiver operating characteristic (ROC) curve and the area under the curve (AUC). The clinical application value of the models was comprehensively evaluated using decision curve analysis (DCA).

**Results:**

The radiomics signature, consisting of 14 selected radiomics features, showed excellent performance in distinguishing between PTMA and LP, with an AUC of 0.90 (95% CI, 0.83–0.96) in the training cohort and 0.88 (95% CI, 0.79–0.97) in the validation cohort. A nomogram model was developed based on the radiomics signature and clinical features. It had a powerful discriminative ability, with the highest AUC values of 0.94 (95% CI, 0.90–0.98) and 0.91 (95% CI, 0.84–0.99) in the training cohort and validation cohort, respectively, which were significantly superior to the clinical model alone. There were no significant differences in calibration curves from Hosmer–Lemeshow tests between training and validation cohorts (*p* = 0.183 and *p* = 0.218), which indicated the good performance of the nomogram model. DCA indicated that the nomogram model exhibited better performance than the clinical model.

**Conclusions:**

The nomogram model based on radiomics signatures of CT images and clinical risk factors could help to differentiate PTMA from LP, which can provide appropriate therapy decision support for clinicians, especially in situations where differential diagnosis is difficult.

## 1 Introduction

Lung cancer is the most commonly diagnosed cancer and the leading cause of cancer death in humans globally (1, 2). The most common lung cancer histologic type is adenocarcinoma. Pneumonic invasive mucinous adenocarcinoma (PIMA) was deemed a new type in the Classification of Lung Adenocarcinoma by the International Association for the Study of Lung Cancer/American Thoracic Society/European Respiratory Society in 2011 (3). PIMA was formerly known as mucinous bronchioloalveolar carcinoma (BAC). PIMA is a relatively rare and specific subtype of adenocarcinoma and accounts for only 2–5% of pneumonic invasive adenocarcinomas (1). In general, PIMA develops insidiously, progresses slowly, and lacks specificity in terms of clinical symptoms and signs. Nevertheless, cough, sputum, hemoptysis, chest tightness, dyspnea, and fever are typical symptoms of PIMA.

On imaging, there are two main types of PIMA: nodular mass type mucinous adenocarcinoma and pneumonic-type mucinous adenocarcinoma (PTMA) (4). The former is difficult to distinguish from common adenocarcinoma on imaging, but misdiagnosis is nevertheless unlikely to occur. PTMA exhibits very similar imaging features (e.g. large lamellar hyperdense shadow) to those of lobar pneumonia (LP) and is therefore readily misdiagnosed as LP. Often, misdiagnosis of PTMA as LP delays its treatment (5). The origin, prognosis, and treatment of PTMA and LP are different, and the prognosis of patients with PTMA is very poor (6). Therefore, prompt and accurate diagnosis of PTMA is essential for patients to receive timely treatment.

By quantifying the regularity and roughness of the gray-scale spatial distribution of pixels in an image, radiomics methods can quantitatively extract texture features and provide a large amount of information about the interior of a lesion that cannot be observed by the naked eye. This non-invasive method has shown its potential usefulness for the identification of internal tumor heterogeneity (7, 8). In recent decades, radiomics has been well proven in the identification, staging, and evaluation of lung cancer (9). Wang et al. found that CT imaging features characteristic of PIMA might provide prognostic information and individual risk assessment in addition to clinical factors (10). Huo et al. have reported that some CT imaging characteristics could be useful in the identification of pneumonic−type lung adenocarcinoma (11). However, there were few studies using radiomics to distinguish PTMA from LP.

Therefore, we conducted a study to identify PTMA and LP based on radiomics, and we summarized the imaging manifestations and corresponding pathological basis. We hope to increase clinicians’ understanding of PTMA, improve treatment outcomes of this disease, and reduce the risk of misdiagnosis. Our study has important implications for the characterization, treatment, and prognosis of this disease.

## 2 Materials and methods

### 2.1 Study design and workflow

**Figure 1:**
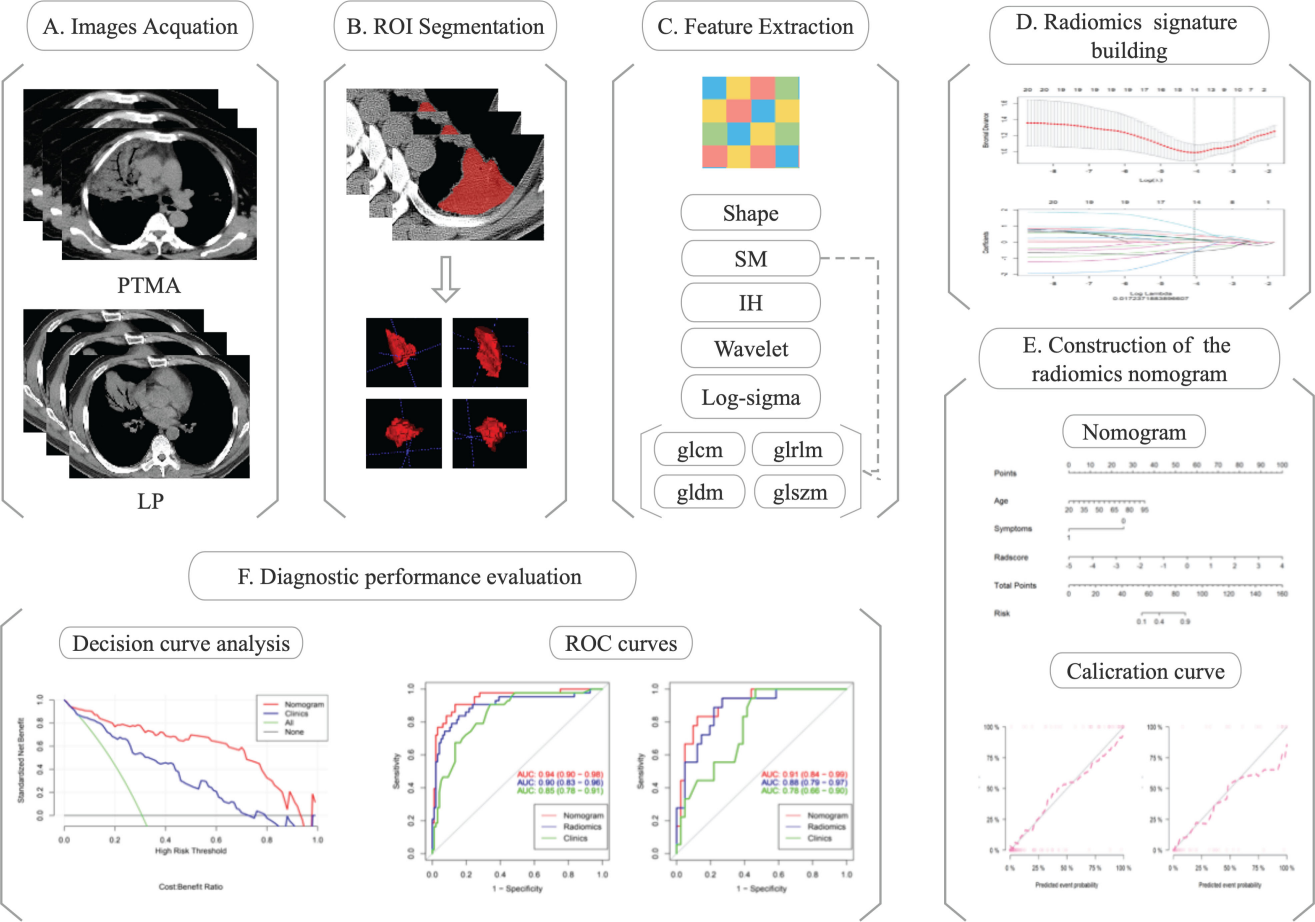
The flow diagram of this study.

Chest CT images of eligible patients (diagnosed with PTMA and LP) were enrolled for radiomics analysis. The regions of interest (ROIs) were delineated along the margin of the lesions. Radiomics features were extracted from ROIs. Radiomics features were selected depending on their efficacy in distinguishing PTMA from LP. Finally, a nomogram model was developed and Rad-scores were evaluated for the training and validation cohorts. The flow diagram of this study is shown in **Figure 1**.

### 2.2 Datasets

This retrospective study was approved by the Research Ethics Committee of the Affiliated Cancer Hospital of Shandong First Medical University, and informed consent was waived. A total of 199 eligible patients diagnosed with PTMA (61 patients) and LP (138 patients) between July 2014 and March 2022 were selected from three tertiary hospitals in Shandong (Affiliated Cancer Hospital of Shandong First Medical University Hospital, The Second Affiliated Hospital of Shandong First Medical University, and Shandong Provincial Hospital) with complete clinical, imaging, and pathological data. Inclusion criteria were as follows: (1) aged over 18 years; (2) standard chest CT scan with clear image quality; (3) diagnosis of pulmonary mucinous adenocarcinoma confirmed by pathology; and (4) PTMA defined as the main manifestation of large lamellar solid shadow on CT. Exclusion criteria were the following: (1) the lesion is too small to extract radiomics parameters effectively; (2) the patient received medical or surgical treatment before CT examination; (3) the patient had received chemotherapy, radiotherapy, or other oncologic therapy before chest CT scans. The 199 patients were assigned randomly to either the training or validation cohort at a ratio of 7:3. The model was developed in the training cohort and evaluated in the validation cohort.

### 2.3 CT image acquisition and processing 

#### 2.3.1 Image acquisition

Chest CT images were obtained using one of four scanners (the Philips Brilliance iCT 128, the Philips CT Brilliance 256, the SOMATOM Definition AS+, and the Philips IQon Spectral CT). Detailed parameters for scanning and reconstruction are listed in **Table 1**. While undergoing the chest CTs, patients maintained the supine position, and the scans were conducted with patients performing end-inspiratory breath holding, covering the entire lung.

**Table 1:** The detailed scan and reconstruction parameters.

Setting	Philips Brilliance iCT 128	Philips CT Brilliance 256	SOMATOM Definition AS+	Philips IQon Spectral CT
Tube voltage (kVp)	120	120	120	120
Tube current (mA)	200	250	200	Auto
Pitch	0.8	0.8	1.2	1.015
Collimation	64×0.625 mm	64×0.625 mm	128×0.6 mm	Auto(64×0.625 mm)
Rotation time	0.5	0.5	0.5	0.5
Slice thickness of reconstruction (mm)	5	5	5	5
Slice interval of reconstruction (mm)	5	5	5	5

#### 2.3.2 Image processing 

Areas with lung lesions are considered to be the ROIs on the lung-mediation window of CT images. Open-source ITK-SNAP software (www.itk-snap.org) was used to manually delineate the ROI along the margin of the lesion by a trained radiologist in chest CT interpretation and then fused into the volume of interest (VOI). Then, VOIs were reviewed slice by slice by another experienced radiologist.

### 2.4 Radiomics feature extraction

Radiomics features were extracted using AK software (AnalysisKit, version 3.2.0, GE Healthcare, China) backend software with the pyradiomics toolkit (version 3.0.1, https://pyradiomics.readthedocs.io/en/latest/) on a Python (Version 3.8.3) platform (12). The images firstly underwent normalization; this involved resampling the voxel size into 1.0×1.0×1.0 mm^3^, discretizing the gray values using 25 bin width and normalizing with the limitation of dynamics to μ ± 3δ (μ: gray level mean; δ: standard deviation). Then, 100 radiomics features were extracted from original CT images, including 14 shape-based features, 18 first-order intensity histogram (IH)-based features, and 68 statistical matrix (SM)-based features, which divided into 22 gray-level co-occurrence matrix-based features, 16 gray-level run-length matrix-based features, 16 gray-level size zone matrix-based features, and 14 gray-level dependence matrix-based features. Moreover, 688 wavelet-based features (including IH and SM features) were extracted from eight wavelet decompositions and 430 log-sigma-based features (including IH and SM features) were extracted from five log-sigma decompositions. All features except the shape features were computed based on the original CT images or Gaussianor wavelet-filtered images.

### 2.5 Radiomics feature selection and radiomics score construction

Radiomics features were selected according to the Maximum Relevance Minimum Redundancy (mRMR) and the least absolute shrinkage and selection operator (LASSO). The mRMR is a method to select the first K features with a high correlation with classification variables, and a low correlation between themselves (13). LASSO was conducted to determine the number of features. The feature subset with the most predictive performance was selected and the corresponding coefficients were evaluated. The radiomics signature (Rad-score) was calculated by weighted summation of the selected features coefficients.

### 2.6 Prediction nomogram build and radiomics validation

Clinical data, including age, gender, symptoms, lobe location, and number of affected lobes, were assessed using univariate and multivariate logistic regression analyses. Univariate analysis was used to assess and find clinical factors with *p*<0.05. The backward step-wised multivariate logistic regression analysis was used to construct the clinical model, with Akaike information criterion (AIC) as the criterion based on the clinical risk factors. Meanwhile, a prediction nomogram model combining the Rad-score and independent clinical risk factors was developed based on multivariate logistic regression. The predictive performance of logistic regression model in the training and validation cohorts were assessed using the receiver operating characteristic (ROC) curve and the area under the curve (AUC). DeLong’s test (14) was used to test the differences of ROC curves between different models. The effectiveness of the nomogram model was assessed by the calibration curve and the Hosmer–Lemeshow test. The net benefit of clinical application of the normogram model was evaluated using decision curve analysis.

### 2.7 Statistical analysis

Statistical analysis was performed using R software (version 4.0.2, www.r-project.org). A *p*-value of < 0.05 represented statistical significance for all two-sided tests.

## 3 Results

### 3.1 Patient characteristics

In total, 199 patients (61 cases of PTMA and 138 cases of LP) fulfilled the inclusion and exclusion criteria. These patients were randomly assigned to either the training cohort (*n* = 140) or the validation cohort (*n* = 59). The detailed clinical and radiological parameters of patients in the training and validation cohorts are presented in **Table 2**. The results in **Table 2** revealed significant differences in age and symptoms, but there were no other significant differences between the two cohorts, including gender, side of the lung, lobe location, and number of affected lobes.

**Table 2:** Clinical and radiological parameters of patients in the training and validation cohorts.

Characteristics		Training cohort (n = 140)			Validation cohort (n = 59)		
		PTMA, n (%)	LP, n(%)	*p*-value	PTMA, n (%)	LP, n (%)	*p*-value
Age (mean ± SD, years)				<0.001*			0.001*
	Mean ± SD	61.2 ± 11.4	44.2 ± 15.6		59.4 ± 10.3	46.0 ± 16.2	
Gender				1.000			0.712
	Female	18 (41.9)	40 (41.2)		10 (55.6)	19 (46.3)	
	Male	25 (58.1)	57 (58.8)		8 (44.4)	22 (53.7)	
Respiratory symptoms				<0.001*			0.045*
	With symptoms	34 (79.1)	96 (99.0)		14 (77.8)	40 (97.6)	
	Without symptoms	9 (20.9)	1 (1.0)		4 (22.2)	1 (2.4)	
Right/left lung				0.660			0.072
	Right	25 (58.1)	55 (56.7)		8 (44.4)	25 (61.0)	
	Left	16 (37.2)	40 (41.2)		8 (44.4)	16 (39.0)	
	Bilateral	2 (4.7)	2 (2.1)		2 (11.1)	0 (0.0)	
Lobe location				0.757			0.379
	Upper	17 (39.5)	37 (38.1)		3 (16.7)	15 (36.6)	
	Middle	4 (9.3)	15 (15.5)		1 (5.6)	4 (9.8)	
	Lower	19 (44.2)	37 (38.1)		12 (66.7)	19 (46.3)	
	≥2	3 (7.0)	8 (8.2)		2 (11.1)	3 (7.3)	
Number of affected lobes				0.313			0.308
	1	39 (90.7)	89 (91.8)		16 (88.9)	38 (92.7)	
	2	3 (7.0)	8 (8.2)		1 (5.6)	3 (7.3)	
	≥3	1 (2.3)	0 (0.0)		1 (5.6)	0 (0.0)	
Case distributions and percentages from three hospitals							
	Hospital 1	38 (19.1)	19 (9.6)		17 (8.5)	7 (3.5)	
	Hospital 2	5 (2.5)	16 (8.0)		1 (0.5)	10 (5.0)	
	Hospital 3	0 (0.0)	62 (31.2)		0 (0.0)	24 (12.1)	

# Comparison between the training cohort and validation cohort;*p< 0.05 two-sample t-test were used for continues variables; χ2 test and Fisher’s exact test were used for categorized variables. SD, standard deviation.

### 3.2 Establishment and validation of the Rad-score

**Figure 2:**
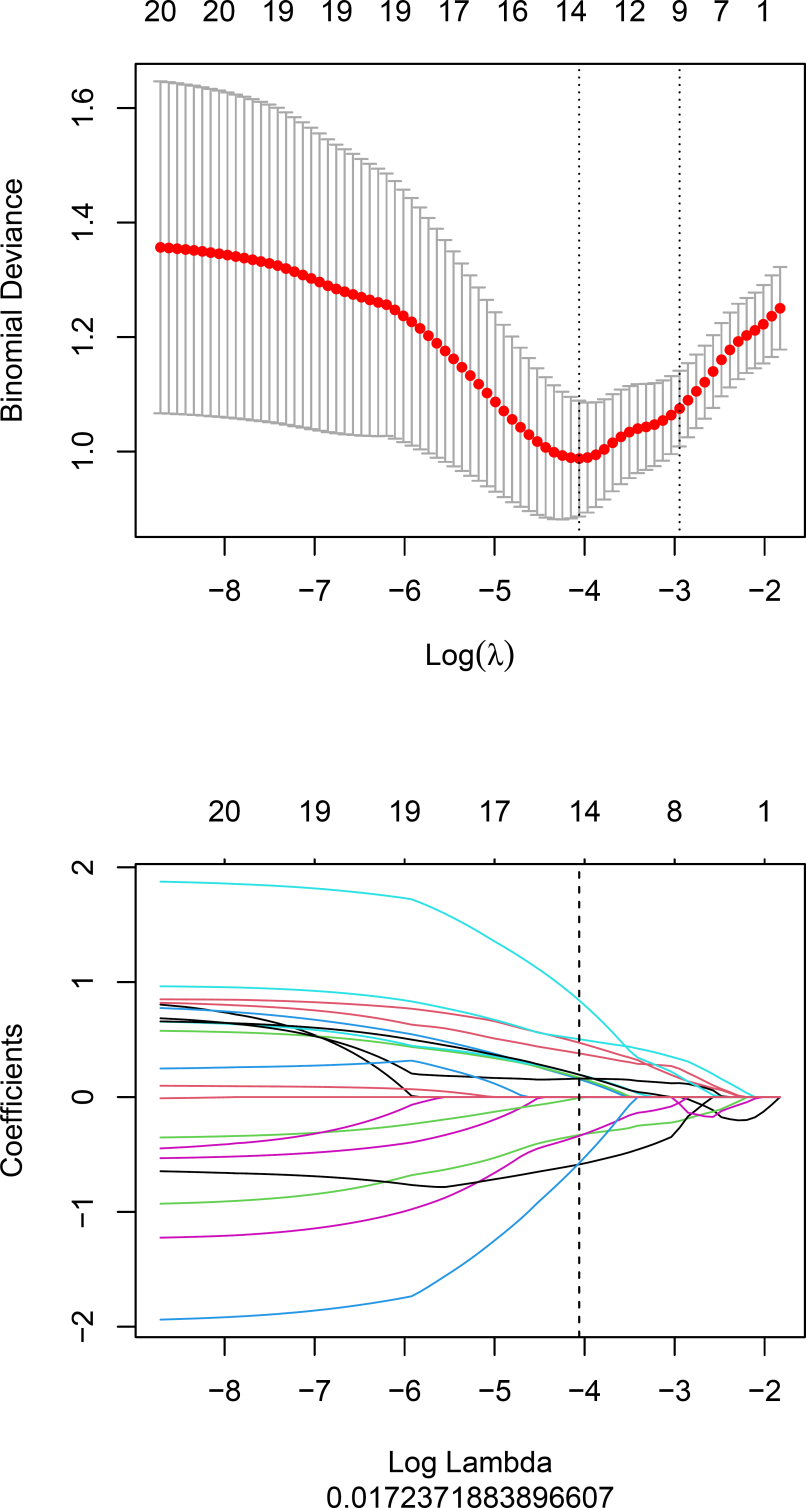
Feature selection for the LASSO logistic regression.

Fourteen radiomics features with non-zero coefficients were retained after feature selection using a LASSO logistic binary regression model (λ = 0.017) (**Figure 2**). The Rad-score was derived from linear combinations of selected prediction features and corresponding coefficients. The following was the formula for the Rad-score:

Rad-score = 0.473*wavelet-LLH_glszm_ZoneEntropy
+–0.33*wavelet-HHL_gldm_LargeDependenceHighGrayLevelEmphasis
+0.156*wavelet-HHL_glszm_LargeAreaLowGrayLevelEmphasis
+0.504*log-sigma-4–0-mm-3D_firstorder_Kurtosis
+–0.338*wavelet-LHH_glszm_ZonePercentage
+–0.587*wavelet-LHH_glcm_MaximumProbability
+0.169*wavelet-HHH_glszm_GrayLevelNonUniformity
+–0.575*wavelet-HHL_glszm_LowGrayLevelZoneEmphasis
+0.196*original_glszm_GrayLevelNonUniformityNormalized
+0.16*wavelet-HHL_glcm_Imc1
+0.38*wavelet-HHH_glcm_ClusterProminence
+–0.008*wavelet-HHL_firstorder_Mean
+0.841*wavelet-HHL_gldm_SmallDependenceLowGrayLevelEmphasis
+0.195*original_shape_Flatness + –1.124

**Figure 3:**
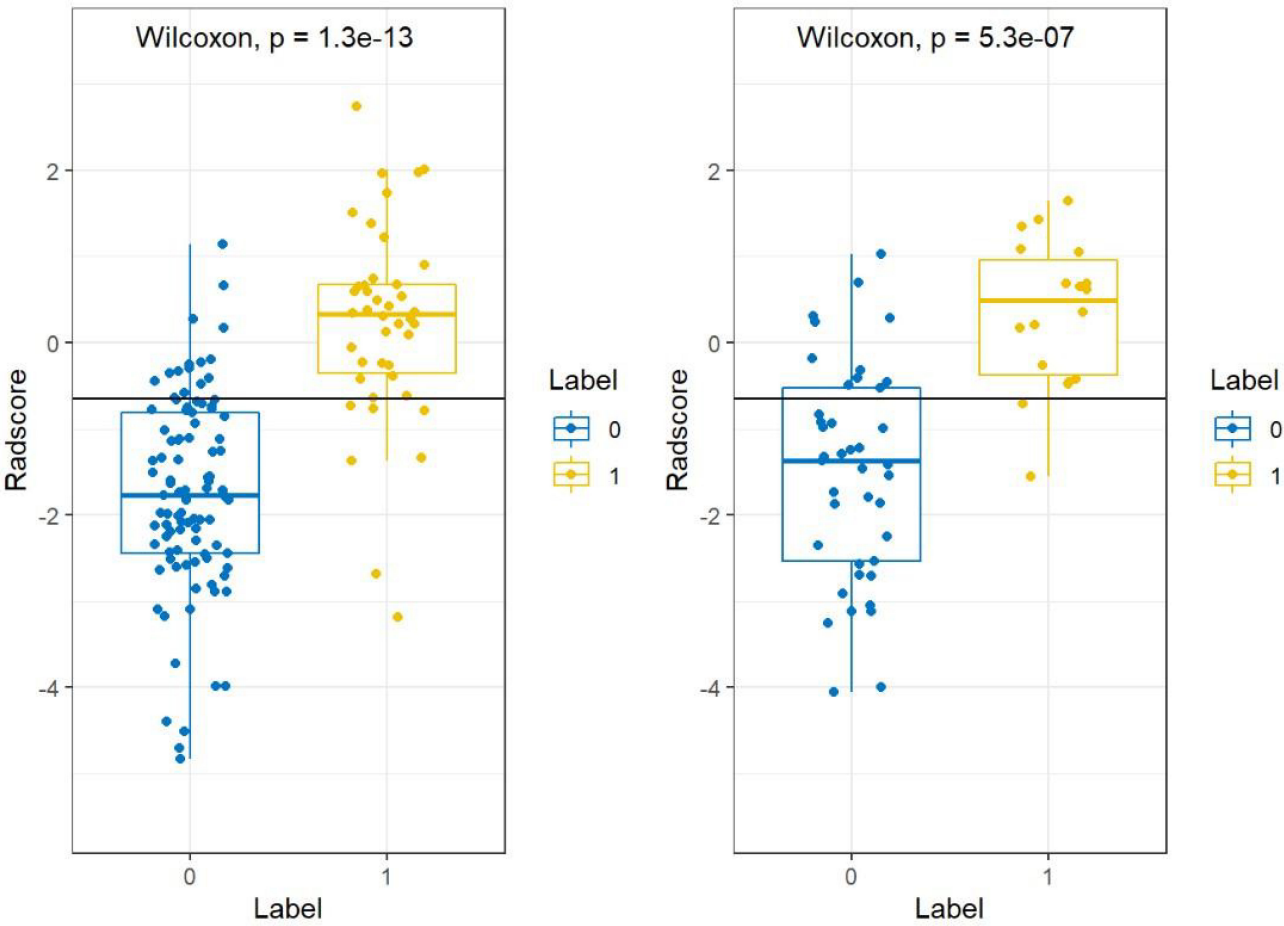
Boxplots between PTMA and LP in the **(A)** training and **(B)** validation cohorts, respectively. *p*-value <0.0001. LP, lobar pneumonia; PTMA, pneumonic-type mucinous adenocarcinoma.

**Figure 4:**
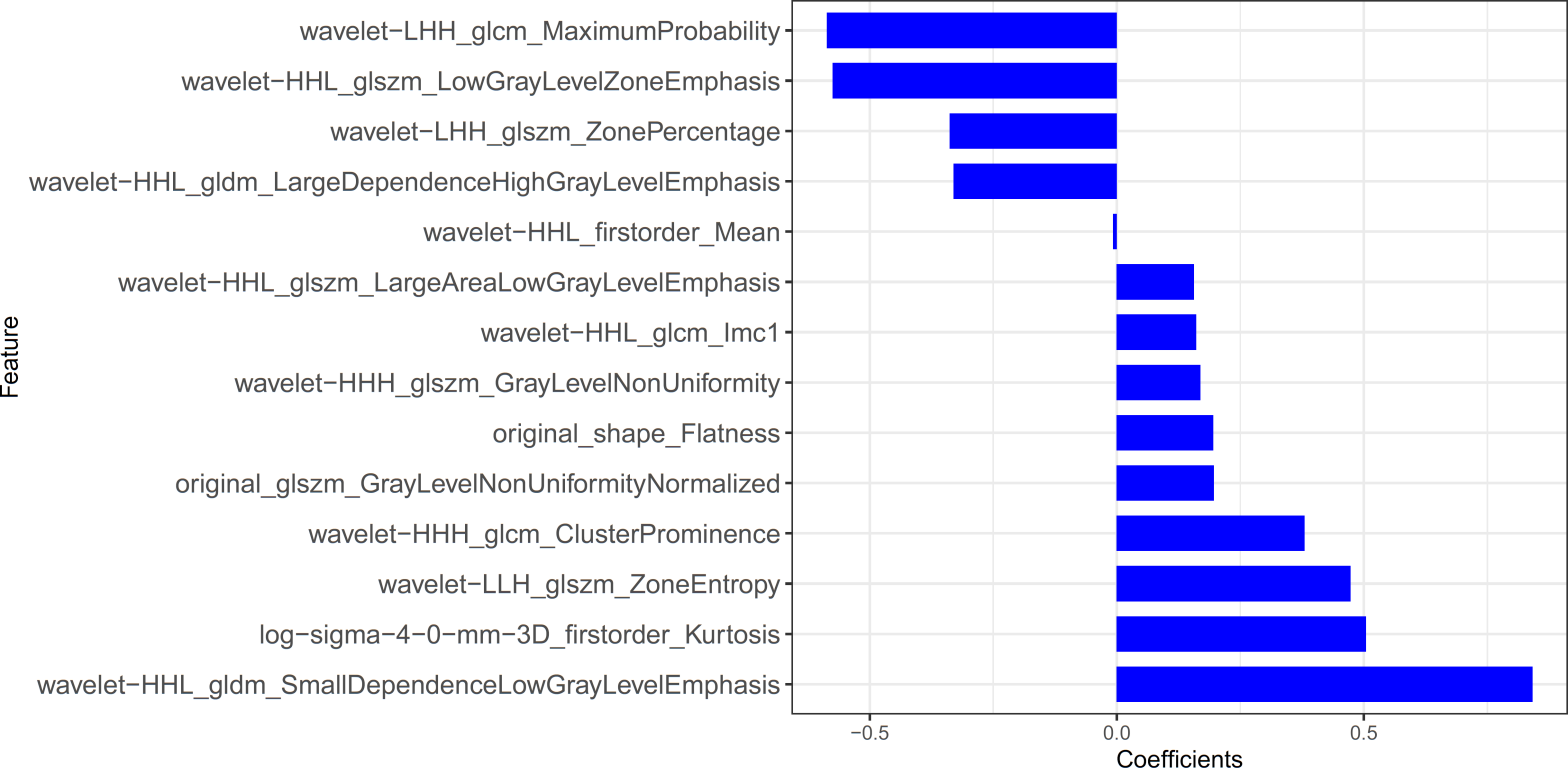
The histogram of the Rad-score: the y-axis indicates the selected fourteen radiomics, and the x-axis represents the coefficient of radiomics.

The statistical distribution of Rad-scores in the training and validation cohorts were illustrated by the plotted box plots (**Figure 3**). The training and validation cohorts had statistically significant differences (all *p*-values were <0.0001). After the number of feature determined, the most predictive feature subset was chosen and the corresponding coefficients were evaluated; the Rad-score was then derived. The Rad-score histogram is in **Figure 4**.

**Figure 5:**
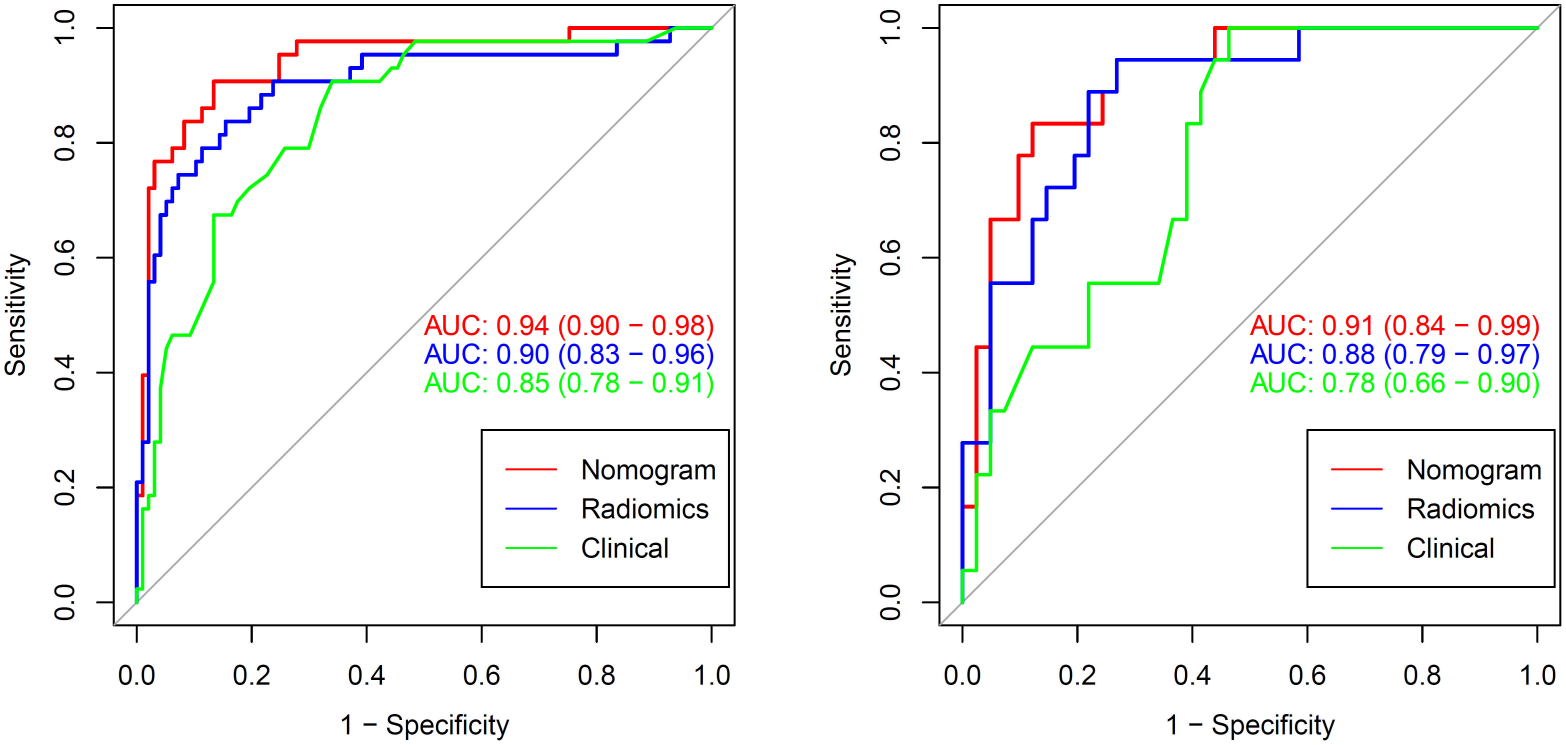
The AUCs of clinical, radiomic and nomogram models in the training cohort and validation cohort. The AUCs of the nomogram models were higher than that of the clinical model and radiomic model in the training and validation cohorts. AUC, area under the curve.

There was a significant difference in the Rad-scores of PTMA and LP patients in the training cohort [0.3 (–0.3, 0.7) vs –1.8 (–2.4, –0.8), *p* < 0.0001], which was also the case in the validation cohort [0.5 (–0.4, 1.0) vs-1.4 (–2.5, –0.5), *p* < 0.0001]. The AUC of the established radiomics model in the training cohort was 0.90 (95% CI, 0.83–0.96), a result similar to the AUC in the validation cohort, which was 0.88 (95% CI, 0.79–0.97). The ROC curves are summarized in **Figure 5**. The accuracy, sensitivity and specificity were 84.29%, 83.72%, 84.54%, and 77.97%, 88.89%, 73.17%, respectively, for PTMA and LP (**Table 2**).

### 3.3 Development of the nomogram model

**Figure 6:**
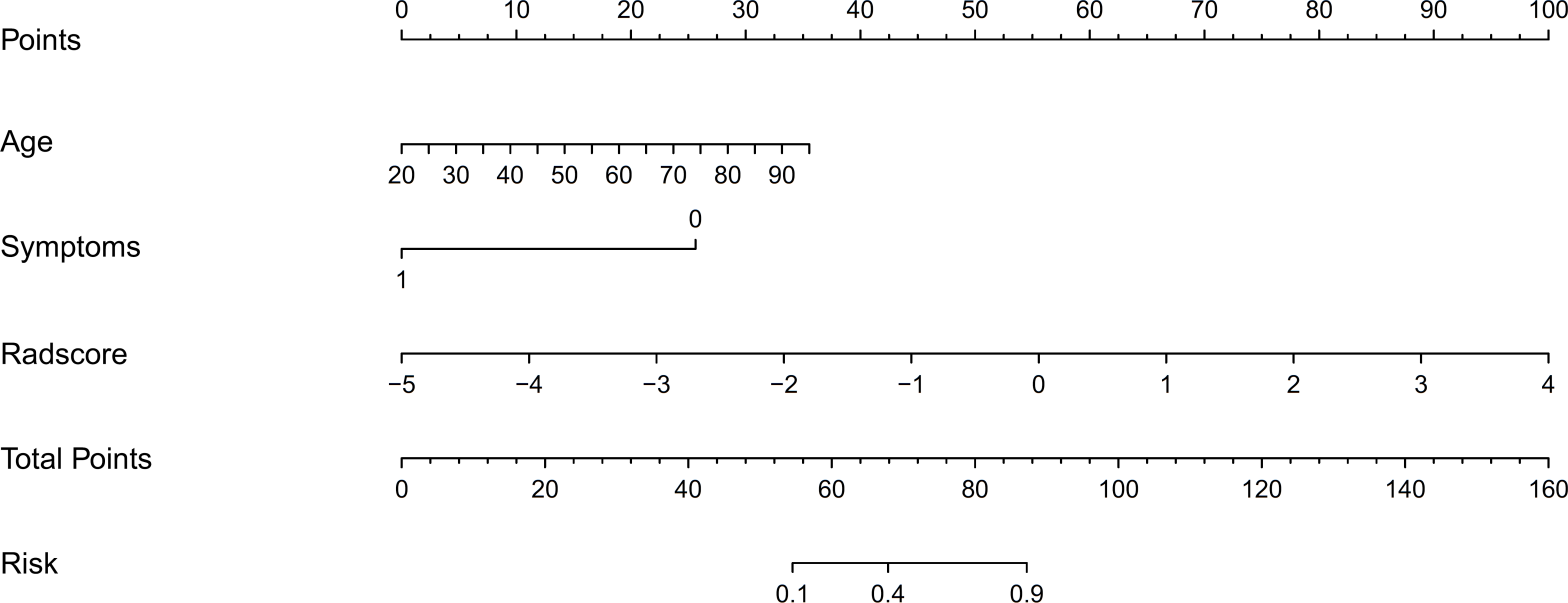
Nomograms constructed in this study using the training cohort.

**Figure 7:**
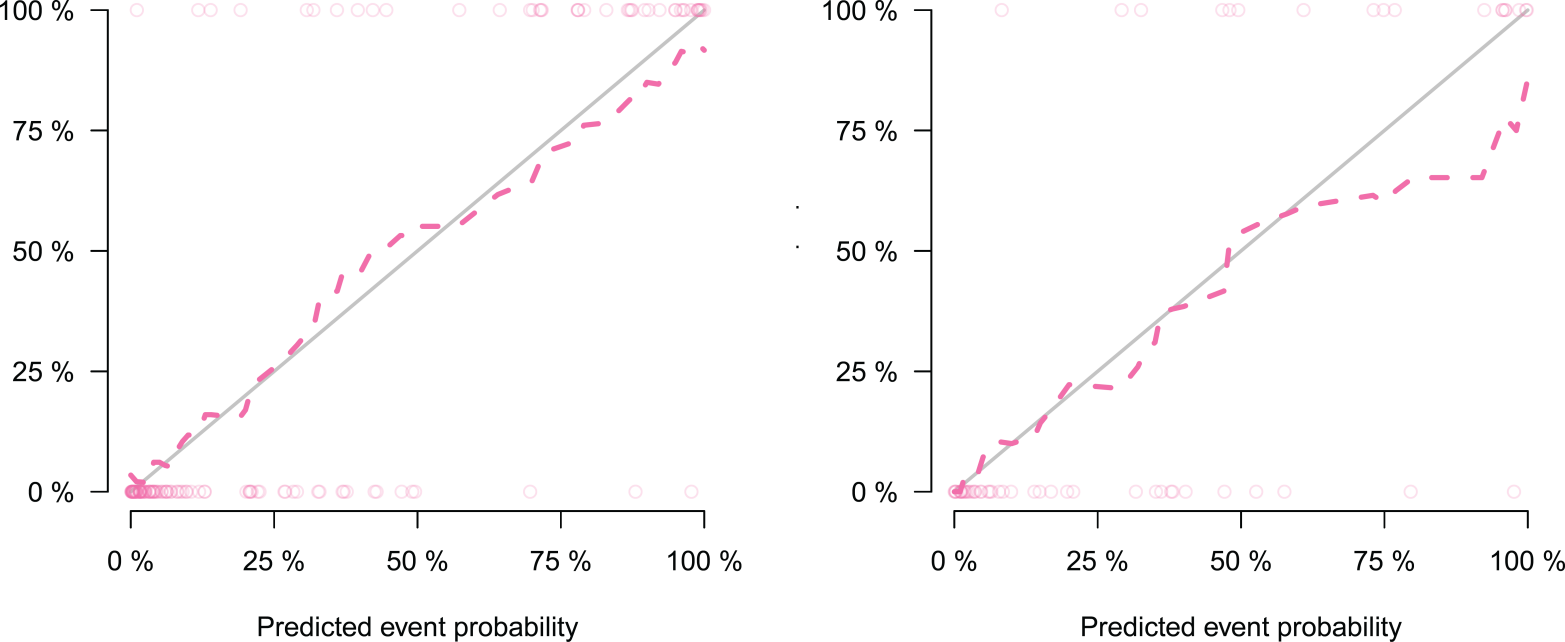
Calibration curve of radiomics nomogram, which showing the relationship between the predicted value and the true value. Left: calibration curve of the training cohort; Right: calibration curve of the validation cohort. The closer the dotted line is to the solid line, the better the predictive power of the model.

We developed a nomogram model based on clinical factors and Rad-scores to reveal the performance for prediction ability of radiomics features, as shown in **Figure 6**. The Nomo-score was calculated as follows: Nomoscore = (Intercept)*0.110+Age*0.064+Symptoms*–3.45+Rad-score*1.49. There were no remarkable differences in calibration curves with the Hosmer–Lemeshow test between the training and validation cohorts (*p* = 0.183 and *p* = 0.218, respectively), as shown in **Figure 7**. The results showed that the AUC of the nomogram model was 0.94 (95% CI, 0.89–0.98) in the training cohort and 0.91 (95% CI, 0.84–0.99) in the validation cohort. The accuracy, sensitivity and specificity were 87.86%, 90.70%, 86.60%, and 77.97%, 59.26%, 93.75%, respectively, for PTMA and LP (**Table 3**, **Figure 5**).

**Table 3:** Diagnostic efficiency of different models in the training cohort and validation cohort.

Model		Accuracy	Accuracy	Accuracy	Sensitivity	Specificity	AUC (95% CI)	*p*-value of Delong-Test	
		(%)	Lower (%)	Upper (%)	(%)	(%)		versus Radiomics	versus Nomogram
Clinics									
	Training	73.57	65.46	80.66	90.70	65.98	0.85(0.78–0.91)	0.243	0.002*
	Validation	64.41	50.87	76.45	72.22	60.98	0.78(0.66–0.90)	0.119	0.012*
Radiomics									
	Training	84.29	77.18	89.88	83.72	84.54	0.90(0.83–0.96)	–	0.076
	Validation	77.97	65.27	87.71	88.89	73.17	0.88(0.79–0.97)	–	0.357
Nomogram									
	Training	87.86	81.27	92.76	90.70	86.60	0.94(0.90–0.98)	0.076	–
	Validation	77.97	65.27	87.71	59.26	93.75	0.91(0.84–0.99)	0.358	–

AUC, area under the curve.

**Figure 8:**
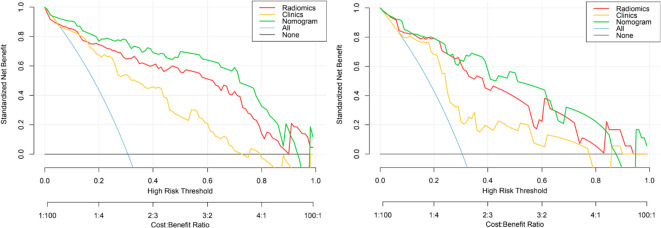
The decision curves of the clinical, radiomics nomogram, and two extreme curves were plotted based on the training and validation cohorts. The decision curves showed that if the threshold probability is > 5%, using a model with the nomogram to distinguish PTMA from LP would be more beneficial than a radiomics model and clinical model. LP, lobar pneumonia; PTMA, pneumonic-type mucinous adenocarcinoma.

Delong’s test showed that the AUC values of the nomogram model were higher than that of the clinical model for the training (*p* < 0.002) and validation cohorts (*p* = 0.012), but not markedly higher than that of the radiomics model, as shown in **Table 2**. There was no obvious difference in the AUC values between the clinical model and radiomics model in the two cohorts. The decision curves (**Figure 8**) also showed that the combined nomogram model provided greater analytical acuity than the clinical model.

## 4 Discussion

**Figure 9:**
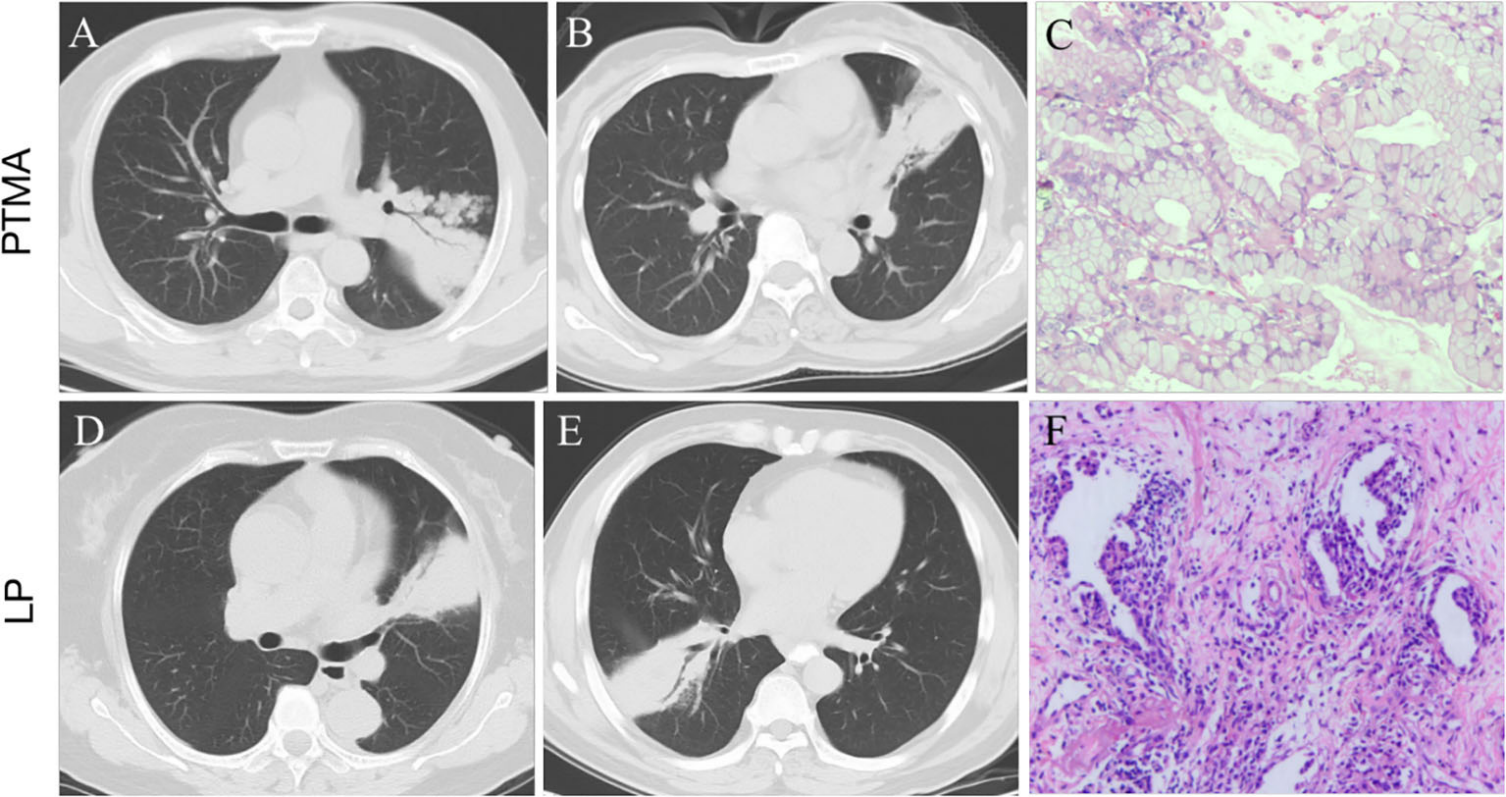
**(A, B)**: pneumonic-type mucinous adenocarcinoma (PTMA) patients with CT scan. **(C)**: Photomicrograph (hematoxylin and eosin staining,×200) confirming invasive mucinous adenocarcinoma with an acinar-predominant pattern. **(D, E)**: lobar pneumonia (LP) patients with CT scan. **(F)**: Photomicrograph confirming chronic inflammatory cell infiltration with fibrous tissue proliferation.

PTMA is defined as an uncommon type of lung cancer with similar imaging features to LP (**Figure 9**) (5, 15). In this retrospective study, 45.9% (28/61) of PTMA patients were misdiagnosed as having LP based on inflammatory lesions on the initial CT scan. It is a challenge to distinguish PTMA from LP by regular CT scans, especially if LP patients do not have typical clinical symptoms or do not respond to anti-inflammatory therapy. Therefore, a method to distinguish PTMA from LP is urgently needed. Most previous studies have been focused on imaging results for pneumonic-type lung adenocarcinoma and/or the relationship between imaging features of pneumonic-type lung adenocarcinoma and survival prognosis (10, 11). There are few studies using radiomics to distinguish PTMA from LP. To enrich the research in this field, we developed and validated a nomogram model based on CT radiomics and clinical festures in differentiating PTMA from LP. The nomogram model had a great performance for the training (AUC = 0.94) and validation cohorts (AUC = 0.91), which confirmed that the nomogram model possessed the potential ability to differentiate between PTMA and LP.

The main CT manifestation of PTMA is a large, lamellar, solid shadow, which is often misdiagnosed as an infectious lesion. There was no significant difference between PTMA and LP in terms of lobe location and number of affected lobes (all *p-* values were > 0.05). Although PTMA has imaging features comparable to LP, there are significant differences in the pathological mechanism and microenvironment the between two groups. Currently, the mechanism of pulmonary adenocarcinoma metastasis is thought to be mediated through the airway (16), which allows tumor cells to grow adherent and metastasize to the distal end of the trachea away from the primary lesion. It has been previously reported that invasive mucinous adenocarcinoma may present on imaging as nodules and masses, or as diffuse exudate, solid lesions resembling pneumonia (17). Different imaging presentations may be associated with different periods of lesion progression (18). Li et al. (19) suggested that the pneumonia type of invasive mucinous adenocarcinoma may be secondary to recurrent inflammatory or associated with mechanized pneumonia.

The pathological type of PTMA is mainly mucinous adenocarcinoma with a histologic growth pattern of adhesive growth. Numerous mucus lakes with floating tumor cells are observed under optical microscopy (5). Furthermore, there are many mucinous adenocarcinoma cells on the residual alveolar walls of the observed mucous lakes. A large amount of mucus is produced by tumor cells, which are dispersed and planted along with the mucus through the airway. Therefore, the tumor gradually develops to multiple lobes, which might lead to the occurrence of PTMA (20–22). With regard to pneumonia, the pulmonary epithelial cells are activated by bacteria or viruses, producing inflammatory mediators that cause damage to pulmonary structures and epithelial cells. These cause the vacuolar degeneration of epithelial cells, swelling of mitochondria (23, 24), intracellular vacuolation, aberration of cytoplasm, and, subsequently, cell damage (25). Furthermore, apoptosis of pulmonary endothelial cells is induced, which eventually leads to pulmonary edema and acute respiratory distress syndrome.

Radiomics, which can extract a large amount of information from images and demonstrate the heterogeneity of lesions (26), is a promising technique developed in recent years (27), which allows for different imaging features to be extracted even from visually unobserved signs. The value of radiomics can be demonstrated especially for lesions that are difficult to identify by the naked eye, which may be the primary advantage of radiomics features in differentiating PTMA from LP. We proved the feasibility of distinguishing PTMA from LP using CT radiomics features. Radiomics features were extracted based on chest CT images, including first-order features, gray level co-occurrence matrix features, gray level run matrix features, gray level size zone matrix features and gray level dependence matrix features. These features can provide high volumes of image details for accurate evaluation of the tumor microenvironment. Finally, we selected 14 non-zero coefficient characteristics to construct the radiomics signature using a LASSO logistic regression model, the results showed that the most predictive characteristics was the wavelet HLL_gldm_SmallDependenceLowGrayLevelEmphasis. The radiomics results revealed the ability of radiomics to distinguish PTMA from LP in the training cohort (AUC = 0.90) and validation cohort (AUC = 0.88).

In this study, we collected clinical and radiological information that may be related to differential diagnoses. Age, gender, respiratory symptoms, and the radiomics signature (lobe location, number of affected lobes) were selected, and a nomogram model combining radiomic and clinical signatures was developed based on these clinical factors. The nomogram model showed a great ability to discriminate between PTMA and LP, and the highest AUCs for the training cohort and validation cohort were 0.94 and 0.91, respectively, which were higher than those of the radiomics model (training cohort: AUC = 0.90; validation cohort: AUC = 0.88) and the clinical model (training cohort: AUC = 0.85; validation cohort: AUC = 0.78). but the difference was The difference was statistically obvious with the clinical model only; this indicates that in terms of AUCs, the nomogram model was superior to the clinical model. The nomogram model did not significantly outperform the radiomics model, but it still had high value in terms of its accuracy (nomogram model and radiomics model, 77.97% vs. 77.97%) in the validation cohort.

Previous studies (28, 29) inferred that radiomics signatures make a remarkable impact on distinguishing lung cancer from inflammatory lesions. We demonstrated the value of quantitative radiomics features for differentiating PTMA from LP. A recent radiomics analysis (30) for classifying focal pneumonia-like lung cancer from pulmonary inflammatory lesions showed that the AUCs were 91.5%, 89.9%, and 80.5%, respectively, in the training, internal and external validation cohorts. In addition, Zhang et al. (31) support the view that radiomic features can assess image heterogeneity; the sensitivity value, specificity value, and AUC for differentiating focal organizing pneumonia from peripheral adenocarcinoma were 0.853, 0.897, and 0.956, respectively. Yang et al. (32) used radiomics based on CT to distinguish solitary granulomatous nodules from solid lung adenocarcinoma in patients with AUCs of 0.935. Feng et al. (33) inferred that radiomic features produced good results for differentiating lung tuberculoma from adenocarcinoma in solitary pulmonary solid nodules by CT scans with AUC = 0.966. Another study (34) demonstrated the great potential of radiomics nomogram to distinguish active pulmonary tuberculosis from lung cancer. Our study is an application of CT radiomics in differentiating PTMA from LP and showed considerable discriminative power.

Image acquisition and lesion segmentation accurately are vital components of radiomics research, which are very significant for feature extraction and model construction. We chose a human manual segmentation method, which is considered the gold standard by radiologists and improves the repeatability, stability, and accuracy of the ROI delineation. The data for our study originated from different CT scanners. However, the effect of different CT scanners on radiomics features has been shown to be limited. For example, Buch et al. (35) suggested that CT texture features rarely correlate with changes in milliampere and kilovolt, and that significant differences in texture features were affected by variations in section thickness. To address these limitations ensured consistency in layer thickness and normalized the images in the present study.

However, our study has some limitations. First, this retrospective study may have been affected by bias in patient selection. Second, although the data for this study originated from different institutions, the number of patients recruited was small; therefore, we did not include the survival analysis of PTMA patients in this study. In future studies, more cases should be collected to verify the differential performance of PTMA and LP. Finally, patients who underwent contrast-enhanced CT were excluded to avoid inconclusive results, but it is unclear whether contrast-enhanced CT affects the accuracy of our results, and this requires further study.

## 5 Conclusions

PTMA and LP have several similarities in terms of clinical symptoms and radiological manifestations, which pose considerable challenges for their clinical diagnosis and treatment. Our results demonstrated that radiomics features on CT scans could be used to distinguish PTMA from LP. Therefore, the nomogram model based on Rad-scores and clinical features could be employed to aid clinicians in making an accurate diagnosis and reduce the risk of misdiagnoses.

## Data availability statement

The raw data supporting the conclusions of this article will be made available by the authors, without undue reservation.

## Ethics statement

This retrospective study was approved by the Research Ethics Committee of the Affiliated Cancer Hospital of Shandong First Medical University, and informed consent was waived. 

## Author contributions

H-JJ designed the research study; Q-QL, Y-XC, and S-Lg acquired the clinical data; M-YG and QC performed the research; H-JJ analyzed the data and wrote the manuscript; J-TZ and S-KN contributed analytic tools; W-HL contributed to revision of the manuscript. All authors have read and approved the final manuscript.
